# The Protective Effect of Apigenin on Myocardial Injury in Diabetic Rats mediating Activation of the PPAR-γ Pathway

**DOI:** 10.3390/ijms18040756

**Published:** 2017-04-04

**Authors:** Umesh B. Mahajan, Govind Chandrayan, Chandragouda R. Patil, Dharamvir Singh Arya, Kapil Suchal, Yogeeta O. Agrawal, Shreesh Ojha, Sameer N. Goyal

**Affiliations:** 1 Department of Pharmacology, R. C. Patel Institute of Pharmaceutical Education and Research, Shirpur, Maharashtra 425405, India; umeshmahajan41@gmail.com (U.B.M.); gvndmshr3@gmail.com (G.C.); pchandragouda@yahoo.com (C.R.P.); 2 Department of Pharmacology, Cardiovascular Research Laboratory, All India Institute of Medical Sciences, New Delhi 110029, India; dsarya16@gmail.com (D.S.A.); kapilsuchal@gmail.com (K.S.); 3 Department of Pharmaceutics and Quality Assurance, R. C. Patel Institute of Pharmaceutical Education and Research, Shirpur, Maharashtra 425405, India; goyalyogita@rediffmail.com; 4 Department of Pharmacology and Therapeutics, College of Medicine and Health Sciences, United Arab Emirates University, P.O. Box, 17666 Al Ain, Abu Dhabi, UAE

**Keywords:** apigenin, streptozotocin, myocardial infarction, diabetic cardiac complications, PPAR-γ

## Abstract

We substantiated the role of peroxisome proliferator-activated receptor-γ (PPAR-γ) activation in the protective effect of apigenin against the myocardial infarction (MI) in diabetic rats. Diabetes was induced by intraperitoneal administration of a single dose of streptozotocin (55 mg/kg). The study groups included diabetic rats receiving vehicle, apigenin (75 mg/kg/day, orally), GW9662 (1 mg/kg/day, intraperitoneally), and a combination of apigenin and GW9662 for 14 days. The MI was induced in all the study groups except the diabetic control group by subcutaneous injection of 100 mg/kg/day of isoproterenol on the two terminal days. The diabetes and isoproterenol-induced MI was evident as a reduction in the maximal positive and negative rate of developed left ventricular pressure and an increase in the left ventricular end-diastolic pressure. The activities of creatine kinase on myocardial bundle (CK-MB) and lactate dehydrogenase (LDH) were also reduced. Apigenin treatment prevented the hemodynamic perturbations, restored the left ventricular function and reinstated a balanced redox status. It protected rats against an MI by attenuating myonecrosis, edema, cell death, and oxidative stress. GW9662, a PPAR-γ antagonist reversed the myocardial protection conferred by apigenin. Further, an increase in the PPAR-γ expression in the myocardium of the rats receiving apigenin reinforces the role of PPAR-γ pathway activation in the cardioprotective effects of apigenin.

## 1. Introduction

Diabetes increases the mortality rate during an acute myocardial infarction (MI) and also contributes to the higher morbidity in the post-infarction patients [1]. Diabetes associated oxidative stress and increased apoptosis contribute to the cardiac co-morbidities of diabetes [2]. Chronic hyperglycemia and advanced glycation end products generate the superoxide anions and reactive oxygen species leading to a greater susceptibility to the cardiovascular complications [3]. An increased susceptibility to the MI and the post-MI morbidity are thus common amongst diabetics. The acute MI related mortality rates in the hospitalized cases and during post-MI care are twice as high for diabetics as for those without diabetes [4]. In diabetics, the therapeutic management of MI mainly relies on the beta blockers, angiotensin converting enzyme inhibitors/angiotensin-II receptor blockers, nitrates, and antithrombotic agents. These drugs reinstate blood supply to the myocardial tissue and prevent the apoptotic damage associated with MI [5]. Despite these therapeutic interventions, the acute MI-induced mortality rate remains more than 30% in diabetics [6].

Diabetic cardiomyopathy associated with the oxidative stress, inflammatory changes, and apoptosis can be modeled in experimental animals [7]. The streptozotocin (STZ)-induced diabetes model in rats is extended to simulate the diabetes associated acute MI by administration of isoproterenol (ISO) [8]. ISO, a non-selective β-adrenoceptor agonist induces MI in rats by a sustained β1 adrenoceptor stimulation that results in an increased lipid peroxidation, generation of free radicals, and exhaustion of the antioxidative defense system. All these isoproterenol-induced physiologic and biochemical alterations cause institution of inflammatory, apoptotic, and necrotic changes in the myocardium and development of an MI. The MI induced by isoproterenol in rats imitates the pathogenesis and characteristic hemodynamic and tissue alterations similar to those observed in the human MI [9].The precipitation of acute MI using isoproterenol in STZ-induced diabetes in rats provides a unique model that could be used to assess the efficacy and the molecular mechanisms of the therapeutic modalities for a diabetes associated acute MI.

Peroxisome proliferator-activated receptor-γ (PPAR-γ), a ligand activated transcription factor regulates numerous biological processes related to the cardiovascular system [10]. It is a member of the nuclear receptor superfamily that is abundantly expressed in the adipose tissues [11]. Experimental studies indicate that the PPAR-γ agonists reduce inflammation and subsequent myocardial injury induced by myocardial ischemia and reperfusion [12,13]. Hence, PPAR-γ appears as a novel therapeutic target to prevent the cardiac complications and heart failure in metabolic disorders [14]. However, in case of diabetic cardiomyopathy the effects of PPAR-γ agonists are diversely reported. In the PROspective pioglitAzone Clinical Trial In macroVascular Events (PRO-active) the PPAR-γ agonist pioglitazone reduced the incidence of cardiovascular complications including non-fatal MI [15]. Conversely, rosiglitazone apparently increased mortality due to cardiovascular complications [16]. This effect of rosiglitazone correlated with its augmenting effects on low-density lipid and cholesterol. Other mechanisms of the PPAR-γ agonists, like alteration of oxidant/antioxidant enzyme activities and attenuation of the systemic inflammation, are proposed to contribute to their cardioprotective effect. The benefits of PPAR-γ agonists outweigh the risks posed by them in treating the cardiovascular complications in diabetics. Therefore, the naturally derived pleiotropic agents modulating PPAR-γ could prove to be novel agents beneficial in the treatment of diabetes associated cardiovascular complications. Among several naturally derived molecules, apigenin (4,5,7-trihydroxyflavone), a flavonoid abundantly present in fruits and vegetables garnered attention for its possible use in cardiovascular and metabolic diseases [17]. Flavonoids significantly modulate the PPAR-γ activity and thereby regulate the glucose/lipid metabolism and inflammatory response. Apigenin reduces the low-density lipoprotein and cholesterol levels [18]. A recent study by Buwa et al., 2015 suggests that the protective effect of apigenin against myocardial injury involves the stimulation of PPAR-γ [17]. Apigenin augments the endogenous antioxidants, favorably modulates the contractile function of the heart, and regulates the death signaling of reactive oxygen species [19]. 

In the present study, we investigated the effects of apigenin on isoproterenol-induced MI in diabetic rats. We substantiated the apigenin mediated PPAR-γ stimulation using a pharmacological challenge approach by administering PPAR-γ antagonist before apigenin treatment.

## 2. Results

### 2.1. Mortality 

The study protocols involved diabetic animals exposed to anesthesia, surgical cannulation of the carotid artery, recording of left ventricular pressure indices after acute and repeated challenges with the isoproterenol. During these procedures, a total of seven animals from the diabetic isoproterenol, diabetic isoproterenol + GW9662, and diabetic isoproterenol + GW9662 + apigenin groups could not withstand the isoproterenol challenge or the subsequent anesthesia and invasive surgical protocol. The mortality during anesthesia and surgery were unpreventable even after careful interventions. The data that was collected from the rats which died during the experiments were excluded from this study. 

### 2.2. General Observations

The diabetic myocardial infarcted rats treated with apigenin alone or in combination with GW9662 showed significant decreases in the blood glucose level when compared with the diabetic isoproterenol group. There was no statistically significant changes in the body weight, heart weight and heart to body weight ratio in the study groups despite of the different treatment schedules (Table 1).

### 2.3. Apigenin Attenuated the Diabetes and Isoproterenol-Induced Alterations in the Electrocardiogram Pattern 

**Figure 1:**
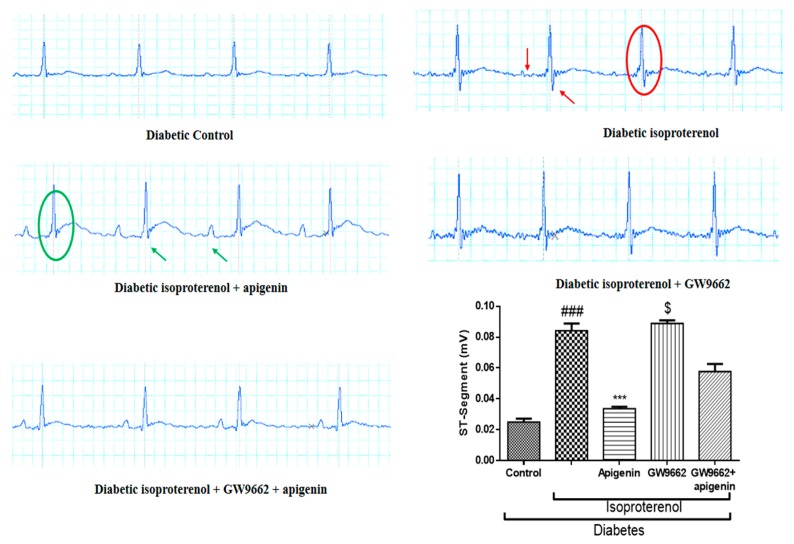
Effect of apigenin on electrocardiogram (ECG) wave forms in streptozotocin (STZ)–isoproterenol-treated rats. The red arrows indicate the deformations of normal ECG waveforms at the place of formation of P-wave and red circle indicates the changes in the QRS complex, especially the ST segment. The green circle indicates the normalization of ECG waveforms and restoration of the ST segment, while the green arrows indicate the normal appearance of the ST segment and P-wave. *** *p* < 0.001 compared to diabetic isoproterenol; ### *p* < 0.001 compared to diabetic control; $ *p* < 0.001 compared to diabetic isoproterenol + apigenin.

The characteristic alternations in the electrocardiogram (ECG) pattern indicating an MI, such as an elevation of the ST segment and rise in the ST height, were observed in the diabetic isoproterenol and diabetic isoproterenol + GW9662 group rats. Apigenin administered orally at a dose of 75 mg/kg/day for 14 days significantly attenuated ST segment elevation and reduced the ST segment height in comparison with the diabetic isoproterenol group rats (Figure 1). PPAR-γ antagonist, GW9662, co-administered with apigenin reverted the ST segment changes and the protection conferred by apigenin treatment was abrogated by GW9662 co-administration.

### 2.4. Apigenin Improved the Hemodynamics and Left Ventricular Function

**Figure 2:**
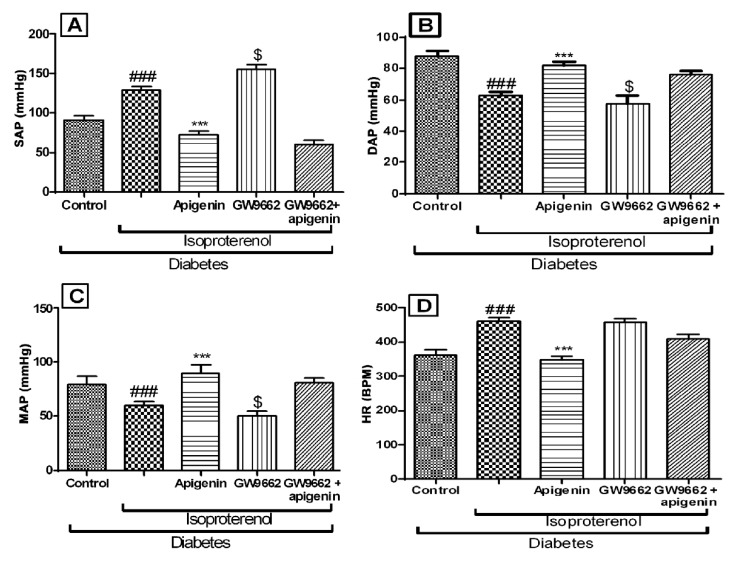
Effects of apigenin on hemodynamic parameters in STZ–isoproterenol-treated rats. (**A**) Systolic arterial pressure (SAP); (**B**) Diastolic arterial pressure (DAP); (**C**) Mean arterial pressure (MAP); (**D**) Heart rate (HR). The data are expressed as mean ± standard error mea (SEM). The significance was determined by one-way ANOVA followed by the Bonferroni’s post hoc test: *** *p* < 0.001 as compared to diabetic isoproterenol; ### *p* < 0.001 as compared to diabetic control; $ *p* < 0.001 as compared to diabetic isoproterenol + apigenin. BPM: Beats per minute.

**Figure 3:**
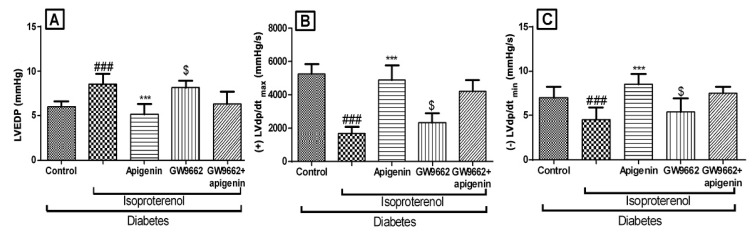
Effects of apigenin on peak positive and negative pressure development in STZ–isoproterenol-treated rats. **(A)** Left ventricular end diastolic pressure (LVEDP), **(B)** Maximal positive rate of left ventricular pressure (+LVdp/dt_max_), **(C)** Maximal negative rate of left ventricular pressure (−LVdp/dt_min_). The data are expressed as the mean ± SEM. The significance was determined by one-way ANOVA followed by the Bonferroni’s post hoc test: *** *p* < 0.001 compared to diabetic isoproterenol; ### *p* < 0.001 compared to diabetic control; $ *p* < 0.001 compared to diabetic isoproterenol + apigenin.

The diabetic isoproterenol group rats showed significantly (*p* < 0.01) lower systolic, diastolic and mean arterial pressure as compared to the diabetic control group. The maximal positive and negative rate of left ventricular pressure (±LVdP/dt_max_) was significantly (*p* < 0.01) reduced in this group. The left ventricular end diastolic pressure (LVEDP) of the diabetic isoproterenol-treated group was found significantly higher than the diabetic control group. All these hemodynamic alterations indicated isoproterenol-induced ischemic changes in the hearts of the diabetic rats. Apigenin treatment was found to improve hemodynamics and left ventricular function as compared to the diabetic isoproterenol-treated group (Figure 2 and Figure 3). GW9662 did not significantly alter the hemodynamics and rather worsened the left ventricular function. These effects of GW9662 were not statistically significant. However, GW9662 significantly countered the protective effects of apigenin on hemodynamics and left ventricular function. 

### 2.5. Apigenin Inhibited Diabetes and Isoproterenol Induced Cardiac Injury 

**Figure 4:**
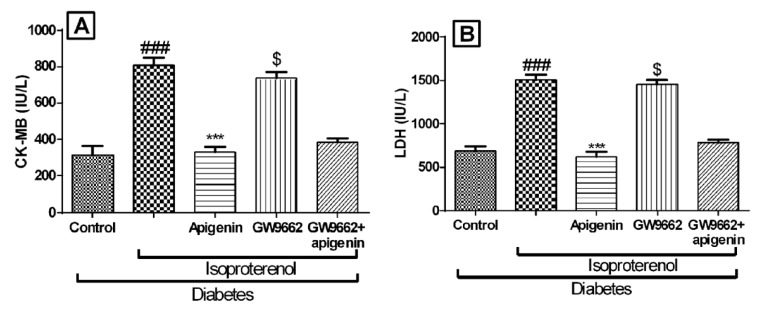
Effects of apigenin on cardiac injury markers in STZ–isoproterenol-treated rats. (**A**) Creatine kinase on myocardial bundle (CK-MB); (**B**) Lactate dehydrogenase (LDH). The data are expressed as mean ± SEM. The significance was determined by a one-way ANOVA followed by the Bonferroni’s post hoc test: *** *p* < 0.001 compared to diabetic isoproterenol; ### *p* < 0.001 compared to diabetic control; $ *p* < 0.001 compared to diabetic isoproterenol + apigenin (IU: International unit).

We determined the effects of different interventions on the cardiac membrane integrity by estimating biochemical markers like creatine kinase on myocardial bundle (CK-MB), and lactate dehydrogenase (LDH). As expected the levels of these markers were significantly reduced in the isoproterenol-treated diabetic rats. Apigenin treatment significantly increased the myocardial levels of CK-MB, and LDH (*p* < 0.001) indicating preservation of the cardiac membrane integrity. The GW9662 pre-treatment inhibited the protective effects of apigenin (Figure 4). The levels of cardiac injury markers in the GW9662 receiving group indicated severe damage to the myocardium. 

### 2.6. Apigenin Improved the Antioxidative Status of Rats Exposed to Diabetic Myocardial Infarction

**Figure 5:**
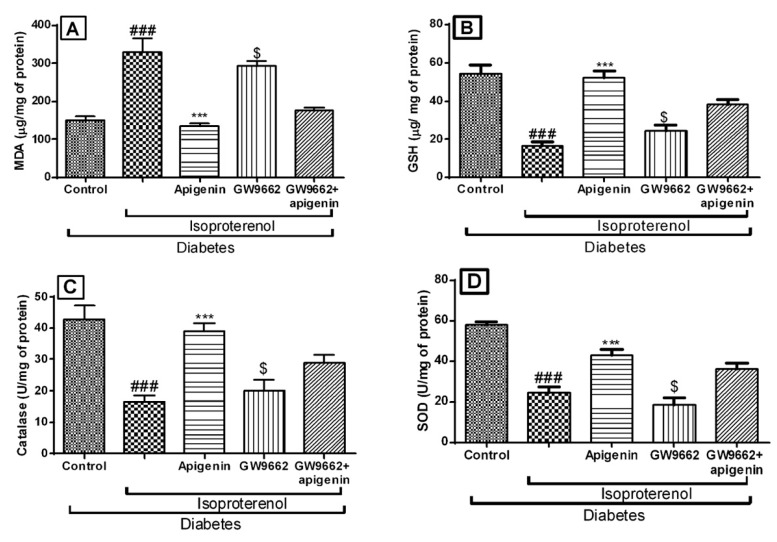
Effects of apigenin on oxidative stress in STZ–isoproterenol treated rats. (**A**) Malondialdehyde (MDA) content; (**B**) Glutathione (GSH); (**C**) Catalase; (**D**) Superoxide dismutase (SOD). The data are expressed as mean ± SEM. The significance was determined by one-way ANOVA followed by the Bonferroni’s post hoc test: *** *p* < 0.001 compared to diabetic isoproterenol; ### *p* < 0.001 compared to diabetic control; $ *p* < 0.001 compared to diabetic isoproterenol + apigenin.

There was a significant (*p* < 0.01) rise in the thiobarbituric acid reactive substances in the diabetic isoproterenol and diabetic isoproterenol + GW9662 + apigenin group rats indicating oxidative damage of the cell membrane. The glutathione (GSH) levels and the activities of superoxide dismutase (SOD) and catalase in myocardial tissue were significantly (*p* < 0.001) reduced. Apigenin treatment inhibited lipid peroxidation and significantly improved the levels of endogenous antioxidants as compared to the diabetic isoproterenol group. As observed in the hemodynamic parameters, the administration of GW9662 abrogated the antioxidant effects of apigenin (Figure 5). 

### 2.7. Apigenin Reduced Bax and Increased Bcl-2 Expression in STZ–Isoproterenol-Challenged Rats

**Figure 6:**
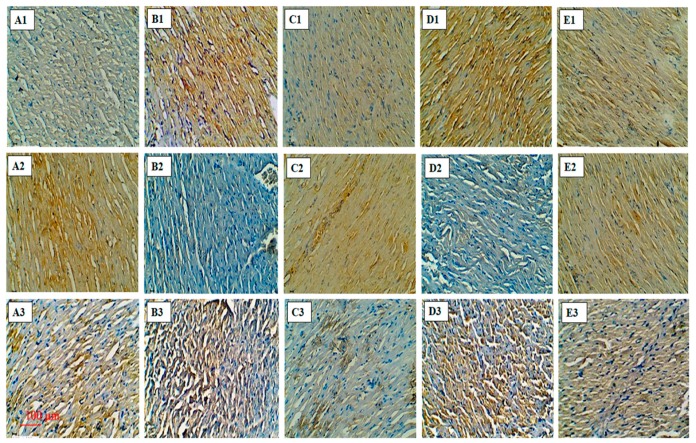
Effects of apigenin on expression of B-cell lymphoma 2 (Bcl-2), Bcl-2 associated X (Bax) proteins and terminal deoxynucleotidyl transferase dUTP nick end labeling (TUNEL)-positive cells in STZ–isoproterenol-treated rats. Immunohistochemical analysis of Bax (**A1**–**E1**) and Bcl-2 (**A2**–**E2**); TUNEL-positive cell (**A3**–**E3**; 20×; Scale bar: 100 µm) in various groups. (**A1**–**A3**): Diabetic control; (**B1**–**B3**): Diabetic isoproterenol; (**C1**–**C3**): Diabetic isoproterenol + apigenin; (**D1**–**D3**): Diabetic isoproterenol + GW9662 (**E1**–**E3**): Diabetic isoproterenol + GW9662 + apigenin.

The diabetic rats treated with isoproterenol showed a significant rise in Bax (B-cell lymphoma 2 (Bcl-2) associated X) and a reduction in the Bcl-2 protein expression. These findings indicate enhanced cell death in the myocardial tissues of the diabetic isoproterenol-treated group. The treatment with apigenin attenuated these alterations in the Bax and Bcl-2 expression. PPAR-γ antagonist; GW9662 alone had no significant effects on the expression of Bcl-2 and Bax. However, when administered with apigenin, GW9662 abrogated the protective effects of apigenin (Figure 6A1–E1,A2–E2). These findings were further supported by the observations of the terminal deoxynucleotidyl transferase dUTP nick end labeling (TUNEL) assay. Apigenin-treated diabetic and isoproterenol-challenged animal’s exhibit significant reduction in the number of TUNEL-positive nuclei as compared to the diabetic isoproterenol group and diabetic isoproterenol + GW9662 + apigenin group. (Figure 6A3–E3). The rats treated with GW9662 alone did not differ from the diabetic isoproterenol group.

### 2.8. Apigenin Increased PPAR-γ Expression in STZ–Isoproterenol-Treated Rats

**Figure 7:**
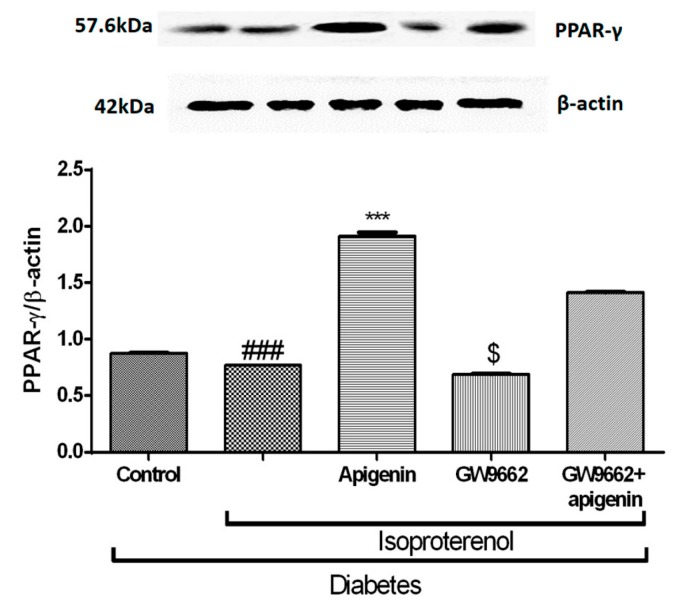
Effects of apigenin on PPAR-γ expression in STZ–isoproterenol-treated rats quantified by Western blot analysis. The data are expressed as mean ± SEM. The significance was determined by one-way ANOVA followed by the Bonferroni’s post hoc test: *** *p* < 0.001 compared to diabetic isoproterenol; ### *p* < 0.001 compared to diabetic control; $ *p* < 0.001 compared to diabetic isoproterenol + GW9662 + apigenin.

The diabetic isoproterenol-treated group showed a significant (*p* < 0.001) reduction in the expression of PPAR-γ in the myocardial tissue. Apigenin treatment significantly (*p* < 0.001) enhanced the PPAR-γ protein expression. The PPAR-γ expression in the diabetic isoproterenol + GW9662 group rats did not differ from the diabetic isoproterenol group. However, when GW9662 was concurrently administered with apigenin, it ameliorated the augmented PPAR-γ expression. This data emphasizes the role of PPAR-γ expression in mediating the protective activity of apigenin in a diabetic MI (Figure 7).

### 2.9. Apigenin Preserved the Myocardial Integrity in STZ–Isoproterenol-Treated Rats

**Figure 8:**
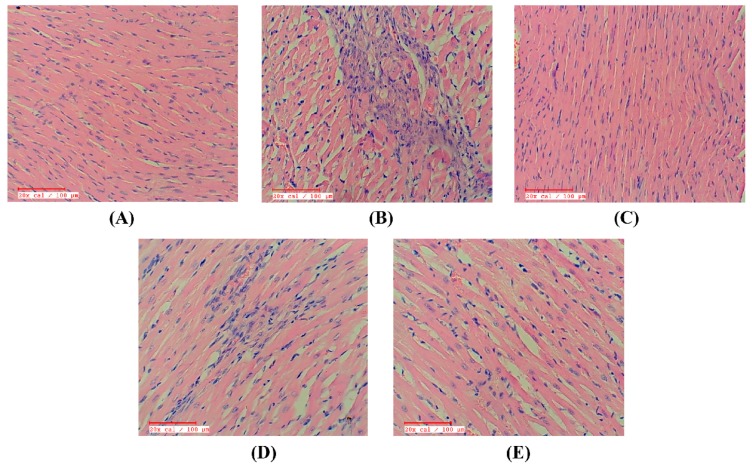
Effect of apigenin on histopathology in STZ–isoproterenol treated rats. (**A**) Diabetic control; (**B**) Diabetic isoproterenol; (**C**) Diabetic isoproterenol + apigenin; (**D**) Diabetic isoproterenol + GW9662; (**E**) Diabetic isoproterenol + GW9662 + apigenin. Scale bar: 100 µm.

The diabetic control rats had normal myocardial structure and architecture with no evidence of edema or inflammation. The myocardial microscopic sections of the diabetic isoproterenol-treated group revealed distinct inflammatory cell infiltrate, cardiac membrane damage, necrosis, and edema in the myocardium. Also, the histological injury score was markedly higher in this group as compared to the diabetic control. However, treatment with apigenin reduced the myonecrosis, preserved myocardial architecture, and had a low histological injury score. The rats receiving GW9662 alone or in combination with apigenin had an increased inflammatory cell infiltrate, membrane damage, myonecrosis, and edema which was reflected as increased histological injury scores (Figure 8). 

## 3. Discussion

The findings of this study indicate that the cardioprotective effect of apigenin involves restoration of cardiac and left ventricular function, maintenance of the endogenous antioxidants, histological salvage of myofibrils, attenuation of apoptosis, and reduction in the lipid peroxidation in the myocardial tissues. In addition, apigenin also enhanced PPAR-γ expression. We ascribe these multipronged efficacies of apigenin responsible for the protection against isoproterenol-induced MI in diabetic rats. This claim can be supported by an observation that the co-treatment with the PPAR-γ antagonist, GW9662, significantly attenuated apigenin mediated cardioprotection. This study provides convincing evidence that the cardioprotective effect of apigenin involves activation of the PPAR-γ pathway. 

PPAR-γ is a nuclear receptor that plays a pivotal role in adipocyte differentiation, glucose homeostasis, and inflammation [20]. The PPAR-γ signaling pathway regulates a variety of biological processes within the cardiovascular system. This nuclear superfamily of transcription factors also suppresses the expression of inflammatory cytokines by controlling the activity of related transcription factors [21]. PPAR-γ agonists reduce the infarct size and inflammation in the ischemia–reperfusion (I/R) model of myocardial infarction [22,23]. However, there are discrepancies in the efficacies of PPAR-γ agonists as protective agents against a MI. Apigenin, a potent agonist of the PPAR-γ is reported to decrease the levels of PPAR-γ via the central pathway of hypoxia-inducible factor (HIF)-1α [24]. 

In the present study, STZ was used for the induction of diabetes in rats. STZ diminishes the activity of nicotinamide adenine dinucleotide (NAD) and reduces the activity of NAD+. It modulates mitochondrial respiratory complexes and thereby reduces the synthesis of ATP. STZ also inhibits the aconitase enzyme activity and causes diabetes in rats [25]. Administration of isoproterenol, a synthetic non-selective β-adrenoceptor agonist in rats is well known to induce myocardial necrosis as a result of imbalance between oxygen supply and demand. Isoproterenol disturbs the physiological equilibrium between the production of free radicals and the antioxidative defense system. It increases the calcium overload in the myocardium, leading to a functional perturbation and associated histological damage. This characteristic alteration in the isoproterenol-induced myocardial injury is similar to a human MI [25].

The diabetic and MI-induced hemodynamic impairment is well demonstrated in the present study as reduced systolic, diastolic, and mean arterial blood pressures, reduced inotropic and lusitropic state (±LVdP/dt_max_) and an increased preload, signified by LVEDP. However, treatment with apigenin decreased LVEDP by increasing the inotropic (+LVdP/dt_max_, indicator of myocardial shriveling) and lusitropic (−LVdP/dt_max_, indicator of reduced myocardial activity) situations of myocardium. On the other hand, PPAR-γ antagonist, GW9662, worsened the hemodynamic condition. Furthermore, prior treatment with GW9662 antagonized the cardioprotective effects of apigenin. The modulation of PPAR-γ confers certain advantages in the treatment of cardiac injury [26]. The observed GW9662-induced reversal of cardioprotective effects of apigenin may at least in part, involve activation of the PPAR-γ pathway.

During an MI, the myocardial membrane integrity is lost and the constituent enzymes, CK-MB isoenzyme, and lactate dehydrogenase of myocardium are released in the serum [27]. In the present study, isoproterenol-challenged diabetic rats showed reduced levels of these enzymes in the myocardium. These effects are in congruence with previous studies [28]. Apigenin treatment restored the activities of these enzymes indicating stabilization of the cardiomyocyte cell membrane. The prior GW9662 administration was found to deplete the activity of these enzymes in the myocardium. This observation can be considered k, to further consolidate the involvement of PPAR-γ in the apigenin conferred protection of the myocardium.

The innate antioxidative substances including SOD, catalase enzymes, as well as GSH defend the myocardium from oxidative stress induced damage [25]. We found that administration of isoproterenol and GW9662 in diabetic rats exhibited a significant decrease in the activities of SOD/catalase enzymes and reduced the GSH levels in the myocardium. These finding indicate an increase in the oxidative stress. Treatment with apigenin significantly improved the activities of antioxidant enzymes and increased the levels of GSH. Apigenin is also known to reduced reactive oxygen species (ROS) production and suppress the oxidative damage to the myocardium in the rat model of MI [17]. Together, these findings provide functional evidence for apigenin induced antioxidant effects may involve activation of the PPAR-γ pathway. 

Lipid peroxidation plays the most crucial and deteriorative role in MI related cellular injury [8]. In the present study, isoproterenol and GW9662 caused a substantial increase in the levels of malondialdehyde (MDA) in the heart due to increased oxidative stress. Elevation of MDA in isoproterenol-induced rats could be attributed to the accumulation of lipids in the heart and permanent damage to the myocardial membranes. Apigenin with or without GW9662 decreased the levels of lipid peroxidation product in the heart of the diabetic myocardial infarcted rats. However, the effect was only more pronounced in the plain apigenin treated group. Thus, this observation helps to put forward one more confirmatory stride in the involvement of the PPAR-γ pathway in the actions of apigenin.

Further, the effect of apigenin on myocardial apoptosis was also studied. Until the recent past, the death of cardiomyocyte in the MI was attributed to necrosis. However, recent findings have proved that apoptosis mainly contributes to such cell death [29]. The expression of pro apoptotic protein Bax and anti-apoptotic protein Bcl-2 were determined by immunohistochemistry. The TUNEL assay was performed for the detection of DNA fragmentation. In the present study, we found a reduction in the Bcl-2 expression and an increase in Bax expression as well as TUNEL-positive cells in the diabetic myocardial infarcted rats. These finding support the role of apoptosis in the death of cardiomyocytes in a diabetic MI. The treatment with apigenin in the diabetic MI rats showed significant anti-apoptotic effect as evidenced by improved Bcl-2 expression and decreased Bax expression as well as reduced TUNEL-positive cells. However, administration of GW9662 aggravated the injuries in the diabetic MI rats. The significance of the PPAR-γ pathway in regulating apoptosis is well established [30]. Thus, an abrogation of the cardioprotective effects of apigenin by GW9662, a PPAR-γ antagonist, supports the role of this receptor in the cardioprotective effects of apigenin. 

Additionally, histopathological studies were performed to reconfirm and support the effects of apigenin in the diabetic MI rats. The rats challenged with STZ and isoproterenol suffered from myonecrosis, edema, and inflammation in the myocardial tissues. Treatment of the diabetic MI rats with apigenin salvaged the myocardium from such histological perturbations. In congruence with other observations, there was a reversal of the effects of apigenin by GW9662 evidenced as edema, inflammation, and myonecrosis. 

## 4. Materials and Methods

### 4.1. Animals

Male Wistar rats weighing 200–250 g were maintained under standard laboratory conditions at 20 ± 2°C and at 55 ± 10% humidity. The rat had free access to water and a standard pellet diet (Nutrivet Life Sciences, Pune, India). The study protocol (IAEC/CPCSEA/RCPIPER/2014-12) was approved by the Institutional Animal Ethics Committee (IAEC) of R. C. Patel Institute of Pharmaceutical Education and Research, Shirpur, India (Committee for the Purpose of Control And Supervision of Experiments on Animals (CPCSEA) registration no. 651/02/C/CPCSEA, Ministry of Environment and Forests, Government of India, New Delhi). All the experimental procedures were conducted according to the guidelines prescribed by the CPCSEA constituted under the Prevention of Cruelty to Animals Act, 1960, India. 

### 4.2. Chemicals

Apigenin, isoproterenol, GSH, SOD, catalase and 1,1,3,3-tetraethoxypropane (standard for MDA estimation) were purchased from the Sigma-Aldrich (St. Louis, MO, USA). The quantitative detection kits for CK-MB isoenzyme and LDH were obtained from Span diagnostics (Mumbai, India). The ABC staining kit and primary antibodies for Western blotting protocols including Bax mouse monoclonal immunoglobulin G2b (IgG2b), Bcl-2/Bax mouse monoclonal immunoglobulin GI (IgGI), PPAR-γ antibody (sc-271392), β-actin (sc-47778) along with the secondary antibodies anti-mouse immunoglobulin G (IgG) were procured from Santa Cruz Biotechnology (Santa Cruz, CA, USA). 

### 4.3. Experimental Protocol

#### 4.3.1. Induction of Diabetes

Diabetes was induced in rats by a single intraperitoneal injection of 55 mg/kg STZ. The STZ solution was prepared in ice-cold 0.1 M cold acidic (pH 4.5) citrate buffer immediately before administration [31]. Rats having serum glucose level above 250 mg/dL on the 3rd day post-STZ injection were selected as diabetic rats for the further study. 

#### 4.3.2. Experimental Design

Diabetic rats were randomized and divided into five different groups each containing 10 rats. The study groups and administered treatments were as follows:

- Diabetic control: This group was orally administered with distilled water as vehicle (1 mL/kg) for 14 days. On 13th and 14th days rats received 0.2 mL saline subcutaneously to serve as vehicle control for isoproterenol injection
- Diabetic isoproterenol-treated: Rats in this group were administered vehicle as above for 14 days along with subcutaneous injections of isoproterenol (100 mg/kg) on the 13th and 14th day
- Diabetic isoproterenol-treated rats administered with apigenin: This group received 75 mg/kg/day of apigenin for 14 days by oral route. On the 13th and 14th day rats were subcutaneously injected with isoproterenol (100 mg/kg)
- Diabetic isoproterenol-treated rats administered with GW9662: Rats in this group received GW9668 (1 mg/kg/day; intraperitoneal (i.p.) injection) for a period of 14 days and subcutaneous injections of isoproterenol (100 mg/kg) on the 13th and 14th days
- Diabetic isoproterenol-treated rats administered with apigenin and GW9662: For 14 days, rats in this group were orally administered with GW9662 15 min prior to the administration of apigenin (75 mg/kg). On the two terminal days of the treatment schedule, isoproterenol (100 mg/kg) was subcutaneously administered to rats in this group

The rats were examined at regular intervals throughout the study period and changes in body weight and/or food and water intake were recorded.

### 4.4. Evaluation of Parameters

#### 4.4.1. Surgical Procedures for Recording Hemodynamic Parameters

The surgical procedure, ECG recording and determination of hemodynamic parameters were carried out as described by Reddy et al., 2015 [32]. The rats were anesthetized with i.p. injection of pentobarbitone sodium (60 mg/kg) and atropine (0.1 mg/kg). The ECG was recorded for 10 min duration following the induction of surgical anesthesia. The systolic, diastolic, and mean arterial blood pressures were determined using MLT844 transducer attached through PE30 cannula surgically introduced into the carotid artery. The same cannula was progressed towards the left ventricle until the thrusts on the ventricular wall were palpated. The parameters including left ventricular end diastolic pressure, maximal and minimum rate of left ventricular functions were recorded through this cannula. 

#### 4.4.2. Estimation of the Cardiac Injury Markers and Oxidative Stress

A 10% homogenate of the heart tissue from each rat was prepared in ice-cold phosphate buffer (50 mM, pH 7.4). The homogenate was centrifuged at 2000 *g* for 20 min at 4°C and the aliquots of the supernatant were used to estimate the extent of the lipid peroxidation, GSH content and activities of catalase, SOD, CK-MB, and LDH. 

#### 4.4.3. Estimation of Lipid Peroxidation

Lipid peroxidation in the heart tissue was determined by measuring the MDA content as described by Goyal et al., 2016 [33]. Briefly, 0.2 mL of the tissue homogenate was mixed with 0.2 mL of 8.1% sodium dodecyl sulfate, 1.5 mL of 30% acetic acid (pH 3.5) and 1.5 mL of 0.8% thiobarbituric acid. The reaction mixture was heated for 60 min at 95°C and then cooled on ice. After cooling, 1 mL of distilled water and 5 mL of *n*-butanol: pyridine (15:1 *v*/*v*) solution was added and the mixture was centrifuged at 5000 rpm using tabletop centrifuge for 20 min. The organic layer was separated and its absorbance was measured at 532 nm. The MDA levels were expressed as µg/mg of protein.

#### 4.4.4. Estimation of Glutathione Content

The GSH content was estimated using the method described by Kumar et al., 2016 [34]. The tissue homogenate (100 µL) was mixed with an equal amount of 10% trichloro acetic acid and then vortexed. The contents were then centrifuged at 5000 rpm for 10 min and 0.05 mL of the supernatant was mixed with 3.0 mL of 0.3 M phosphate buffer (pH 8.4) and 0.5 mL of 5,5′-Dithiobis(2-nitrobenzoic acid). The optical density of the mixture was measured spectrophotometrically at 412 nm within 10 min. The levels of GSH were expressed as μg/mg of protein.

#### 4.4.5. Estimation of Catalase Activity

The catalase activity was estimated by the method described by Sah and Nagarathana, 2016 [35]. A mixture of 1.0 mL of 50 mM phosphate buffer (pH 7) and 0.1 mL of 30 mM hydrogen peroxide was added to 50 µL of tissue supernatant. The kinetics of the decrease in the optical density of the sample was read at 240 nm for 30 s at an interval of 5 s. The activity of catalase was expressed as U/mg of protein.

#### 4.4.6. Estimation of Superoxide Dismutase Activity

The SOD activity was determined by the method described by Hassan, 2015 [36]. Briefly, to 25 µL of tissue supernatant a mixture of 100 µL of 500 mM Na**_2_**CO**_3_**, 100 µL of 1 mM ethylenediaminetetraacetic acid (EDTA), 100 µL of 240 µM/ nitro-blue tetrazolium (NBT; Sigma-Aldrich, St. Louis, MO, USA), 640 µL of distilled water, 10 µL of 0.3% Triton X 100, and 25 µL of 10 mM hydroxylamine was added. The readings were recorded spectrophotometrically in kinetic mode at intervals of 1 min up to 3 min at 560 nm. The enzyme activity was expressed as U/mg of protein.

#### 4.4.7. Determination of Myocardial Apoptosis

##### Immunohistostaining for the Expression of Bax and Bcl-2 Proteins

The myocardial tissue sections were deparaffinised with xylene to remove excess paraffin. The sections were incubated with blocking buffer for 10 min at room temperature. Further, they were incubated overnight at 4°C with primary mouse Bax and Bcl-2 monoclonal antibodies. The tissue sections were incubated for 1 h with biotinylated goat secondary antibody. Subsequently, the sections were washed and incubated for 30 min with peroxidase-conjugated streptavidin-biotin complex. The target proteins (Bax/Bcl-2) were visualized after incubation in peroxidase substrate using 3,3′-diaminobenzidine as the chromogen and the sections were counterstained with haematoxylin and analyzed using light microscopy (Motic AE2000 inverted microscope, Motic microscope, Kowloon Bay, Kowloon, Hong Kong, China) [37].

##### TUNEL Assay

The TUNEL assay was performed using a cell death detection kit (Bio Vision Inc., Milpitas, CA, USA) according to the manufacturer’s instructions. 

#### 4.4.8. Western Blot Analysis

Myocardial tissue was homogenized in radioimmunoprecipitation assay (RIPA) lysis buffer and the protein was estimated. Western blot analysis was performed as described previously [37]. Briefly, the myocardial tissues were separated by sodium dodecyl sulfate–polyacrylamide gel electrophoresis (SDS–PAGE) and transferred to nitrocellulose membrane that was blocked for 2 h with 5% bovine serum albumin. Further, it was incubated for 12 h at 4°C with a rat primary antibody (β-actin and PPAR-γ). The blots obtained were scanned densitometrically to quantify the expression of β-actin and PPAR-γ using Bio-Rad Quantity One 4.4.0 software (Bio-Rad, Hercules, CA, USA).

#### 4.4.9. Light Microscopic Evaluation

Myocardial tissue was fixed in buffered formalin solution and embedded in paraffin. The serial sections (3 µm thick) were cut using a microtome (Leica CM1100, Leica biosystems, Buffalo Grove, IL 60089, USA). Each section was stained with haematoxylin and eosin (H&E), examined under the light microscope, and digital images were captured. The pathologist performing the microscopy was blind tothe treatment and experimental groups.

#### 4.4.10. Statistical Analysis

The data are expressed as mean ± SEM. The statistical significance was analyzed by one-way ANOVA)followed by Bonferroni’s post hoc test using a graph pad prism software, version 6.0, (Fay Avenue, Suite 230, La Jolla, CA, USA). The value of *p* < 0.05 was considered statistically significant. 

## 5. Conclusions

The findings of the current study indicate that apigenin exerts cardioprotective effect by activating the PPAR-γ pathway. The activation of PPAR-γ by apigenin could be considered as a novel approach in the treatment of the diabetes-related cardiovascular complications. Further exploration of natural activators of PPAR-γ could yield more promising druggable molecules with lesser risks of congestive heart failure, weight gain, and carcinogenesis as compared to the synthetic agonists of PPAR-γ. 

## Figures and Tables

**Table 1:** Effect of apigenin on blood glucose and weights in different experimental groups.

Groups	HW (g)	Blood Glucose (mg/dL)	BW (g)	HW/BW Ratio (mg/g)
Diabetic control	0.62 ± 0.03	420.8 ± 53.90	235 ± 3.61	2.72 ± 0.008
Diabetic isoproterenol	0.59 ± 0.08	406.5 ± 54.87	217 ± 3.32	2.71 ± 0.024
Diabetic isoproterenol + apigenin	0.61 ± 0.04	310.3 ± 31.24 ***	239 ± 2.82	2.51 ± 0.014
Diabetic isoproterenol + GW9662	0.56 ± 0.07	424.3 ± 47.24	209 ± 3.41	2.39 ± 0.020
Diabetic isoproterenol + GW9662 + apigenin	0.55 ± 0.04	326.7 ± 24.31 **	228 ± 4.21	2.41 ± 0.009

The data are expressed as mean ± standard error mean (SEM) (*n* = 10). The significance was determined by one-way analysis of variance (ANOVA) followed by the Bonferroni’s post hoc test. ** *p* < 0.05, *** *p* < 0.001 compared to diabetic isoproterenol. BW: Body weight; HW: Heart weight; HW/BW ratio: Heart weight to body weight ratio.
